# Blood Particle Separation Using Dielectrophoresis in A Novel Microchannel: A Numerical Study

**DOI:** 10.22074/cellj.2020.6386

**Published:** 2019-10-14

**Authors:** Omid Zahedi Siani, Mahdi Sojoodi, Mohammad Zabetian Targhi, Mansoureh Movahedin

**Affiliations:** 1. Faculty of Mechanical Engineering, Tarbiat Modares University, Tehran, Iran; 2. Faculty of Electrical and Computer Engineering, Tarbiat Modares University, Tehran, Iran; 3. Faculty of Medical Sciences, Tarbiat Modares University, Tehran, Iran

**Keywords:** Biomedical Applications, Blood Cells, Microfluidics

## Abstract

**Objective:**

We present a four-branch model of the dielectrophoresis (DEP) method that takes into consideration the inherent properties of particles, including size, electrical conductivity, and permittivity coefficient. By using this model, bioparticles can be continuously separated by the application of only a one-stage separation process.

**Materials and Methods:**

In this numerical study, we based the separation process on the differences in the particle sizes. We used the various negative DEP forces on the particles caused by the electrodes to separate them with a high efficiency. The particle separator could separate blood cells because of their different sizes.

**Results:**

Blood cells greater than 12 μm were guided to a special branch, which improved separation efficiency because it prevented the deposition of particles in other branches. The designed device had the capability to separate blood cells with diameters of 2.0 μm, 6.2 μm, 10.0 μm, and greater than 12.0 μm. The applied voltage to the electrodes was 50 V with a frequency of 100 kHz.

**Conclusion:**

The proposed device is a simple, efficient DEP-based continuous cell separator. This capability makes it ideal for use in various biomedical applications, including cell therapy and cell separation, and results in a throughput increment of microfluidics devices.

## Introduction

The field of microfluidics, microfabrication, and research on miniaturized fluidic systems has been developed by 4 main factors: genesis microanalytical methods, microelectronics industry, an expansion of genomic sciences in the 1980s, and development of microfluidic systems for use in biological and chemical weapons in the 1990s (1). For the first time, researchers used microfluidics in the lab-on-a-chip (LOC) devices at the beginning of the 1990s (2). LOC has the capability to run various functions such as microparticle separation and sorting, cell culture, and analysis. An important application of LOC microfluidic devices is the analysis of blood cell components for subsequent applications. Microfluidic devices are used in the chemical industry, to separate micron size objects, mineral processing, biological researches, and diagnostics processes (3). Studies of diagnostics processes have focused on separation of dead cells from living cells and cancer cells from healthy cells.

The mechanical properties and connections among cell structures are important factors for sorting and separation of certain cells from other cells. It has been proven that some diseases such as malaria and gastrointestinal tumours change the mechanical deformability of the cell. Thus, cells can be separated by the use of mechanical properties such as elastic modulus and cell size (4). For example, normal red blood cells pass through blood capillaries; however, those cells infected by malaria parasites cannot circulate in the bloodstream because they are approximately 50 times harder than healthy blood cells, which leads to blocking of the capillaries (5). Similarly, epithelial cancer cells have different physical properties compared with healthy cells.

Cell separation techniques that use microfluidics can be divided into 3 categories: passive, active, and combined. Examples of passive techniques applied for cell or microparticle separation include pinched flow fractionation (6), inertia and dean flow fractionation (7), micro vortex manipulation (8), deterministic lateral displacement (9), hydrodynamic filtration (10), and microhydrocyclone (11). Examples of active techniques include dielectrophoresis (DEP) (12), and magnetic (13), optical (14), and acoustic techniques (15). In general, active methods use an external force field in their performance while passive methods do not use the external force field. The structure of devices that use passive techniques are simpler than active techniques, but the throughput of active techniques is more. The devices that use passive techniques are usually massive.

Table S1 (See Supplementary Online Information at www.celljournal.org) compares some of the cell sorting and isolation methods. Both electrical and acoustic methods have advantages of strong controllability, high efficiency, ease of operation, slight damage, and low cost when compared to other methods. These methods are suitable for the separation and sorting of bioparticles. In this research, we have attempted to manipulate human blood cells using the DEP method.

In the early 20^th^ century, researchers first discussed the DEP phenomenon. In 1951, Pohl (16) studied the separation of solid particles from a polymer solution that resulted from polarization forces produced by the activation of an inhomogeneous electric field, which they named "dielectrophoresis". With the development of micromachining technologies and microelectromechanical systems at the beginning of the 1990s, researchers integrated electrochemical functions into chips, which enabled manipulation of cells, microparticles, and nanoparticles (2). Kim et al. (17) positioned 2 sets of electrodes at different angles for dielectrophoretic cell separation. They tagged target cells with DEP tags (polystyrene beads) and conducted the buffer and sample to the microchannels. In this way, they used a multi-target DEP activated cell sorter (MT-DACS) chip to separate tagged bacterial cells from non-target cells with a high purity. In 2011, Piacentini et al. (18) proposed focusing the particles on one side of the main microchannel using a buffer solution. Platelets were separated from red and white blood cells based on their size difference using a single-stage separation with a nonuniform electric field generated by ‘liquid electrodes’. This resulted in a purity of 98.8%. In 2013, Li et al. (19) converted the electric field generated by a DC power supply to a local nonuniform electric field in an S-shaped curved microchannel by using both a microchannel curvature and obstacles. In this method, 10 and 15 polystyrene particle suspensions diluted by deionized water were injected into the inlet microchannel, and resulted in a successful, efficient particle separation. By the end of 2014, Lewpiriyawong and Yang (20) aligned 5 polydimethylsiloxane (PDMS) insulating blocks along one side of a main microchannel to induce a nonuniform electric field. Then, the buffer solution and samples that comprised one type of fluorescent particle and 2 types of nonfluorescent particles were injected into the microchannel inputs. Cell mixtures of 3 sizes (2 μm, 5 μm, and 10 μm) were separated at a separation efficiency of 99%. In 2016, Ye et al. (21) developed a device that applied the DEP method to separate samples, including the 3 μm, 10 μm, and 25 μm polystyrene microparticles. The multiple particles were separated continuously using a pair of acupuncture needle electrodes embedded in a PDMS as a hurdle in the microchannel, which resulted in more than 90% separation purity. In 2018, Kale et al. (22) presented a DEPbased device that manipulated latex beads with a diameter of 5 μm. The performance of the latex beads was evaluated as a function of the applied voltage.

After an investigation of the presented previous works, we have developed a relatively simple and efficient model. This model is based on the insulator-based DEP method, where the electric field gradient is produced by the applied alternating electric current to the electrodes. The induced force generated by the gradient electric field results in separation of particles with different sizes. For this purpose, the applied alternating voltages to electrodes are approximately 50 V and -50 V. As a result of the hydrodynamic pressure difference between the input and output zones of the main microchannel, the fluid flows in the microchannel and the fluid driving force is applied to the cells. The DEP forces depend on the size of cells. Both the DEP forces and fluid driving force are simultaneously applied to the cells, which causes them to separate. Therefore, by taking into consideration cell size, the cells are moved into the different branches in the downstream main microchannel.

This work has major advantages compared with previous works. In prior works, the multi-branch model of the DEP method was used to separate the latex particles and other test particles, and the separation of blood cells using this method is offered. In the current study, we have used the simple, efficient electrode structure to separate blood cells based on their size. In the current work, separation of the platelets from the white blood cells (large lymphocytes and neutrophils) and red blood cells is a continuous, one-step process done by the ACDEP technique, in contrast to multiple steps used in most of the previous works. Previous studies have used a maximum of 3 branches for the multi-branch model of the DEP method to separate the blood cells. However, if these models could lead to deposition of the blood cells with sizes greater than 12 μm. Offering a four-branch model of the DEP method, resulting in the capability of the proposed design to separate the blood cells with sizes greater than 12 μm using the fourth branch. This could improve separation efficiency and prevent deposition of the particles in the other branches. In the present research, we considered both large lymphocytes and neutrophils to separate the white blood cells from the other blood cells. The neutrophils are the most common white blood cell type, and lymphocytes are the second most common in most mammals (23). The most recent studies have only separated one type of white blood cell from the other blood cells. With regards to the various sizes of white blood cells, it seemed necessary to take both types of white blood cells into consideration for a more accurate separation.

## Materials and Methods

### Dielectrophoretic force exerted on spherical particles

In this numerical study, we considered a variable electric potential with an angular frequency, ω, to produce an alternating electric field (AC). The alternating electrical potential is defined as:

[1] ∅(x,t)=Re[∅-(x)ejωt]

Where: Re[] is the real part of the complex variable (∅), ω is the angular frequency, t represents time, j is defined as: j≡√(1), x is the position vector, and the symbol ∅ represents the potential phasor, regarding the relationship between electric potential and field, that is (24):

[2] $$\overset{-}{E}=\mathrm{-\nabla}\overset{-}{\emptyset }$$

The alternating electric field is obtained:

[3] E(x,t)=Re[E-(x)ejωt]

In [3], E is the electric field phasor. The time-averaged DEP force on the spherical particles in an alternating electric field is calculated as below (24, 25):

[4] 〈FDEP〉=2πεmRe[fcm]R3∇Erms2

Where:‹› indicates the time-averaged DEP force, E_rms_ represents the root-mean-square magnitude of the alternating electric field, ε_m_ is the electric permeability coefficient of the medium, and f_cm_ is the Clausius-Mossotti factor, which is defined as follows (24, 25):

[5] fcm(ε~m,ε~p)=(ε~p-ε~m)(ε~p+2ε~m)

Where: p indicates the particle, m represents the suspending medium, and $\tilde{\varepsilon }$ is the complex permittivity, which is defined as (24):

[6] ε~=ε-J(σω)

Where: σ is electrical conductivities and ω is angular frequency. According to the dielectrophoretic force formula, the following points are notable in the numerical study of particle separation: i. The dielectrophoretic force is a nonlinear function because it is proportional to the square of the electric field, ii. The dielectrophoretic force is proportional to the cube of the particle radius (F_DEP_ has the potential to separate the particles from each other based on the difference in volume), and iii. The dielectrophoretic force is proportional to f_cm_, and the Clausius-Mossotti factor is proportional to ${\tilde{\varepsilon }}_{p}$ and ${\tilde{\varepsilon }}_{m}$. Therefore, F_DEP_ can distinguish particles and cells based on their complex permittivity.

Inserting equation [6] into [5], results in:

[7] fcm=((εp-εm)+jω(σp-σm))((εp+2εm)+jω(σp+2σm))

Which indicates that in the high angular frequency limit, the Clausius-Mossotti factor depends on the dielectric permittivity of the suspending medium and the particle as:

[8] lim(ω→∞)fcm=(εp-εm)(εp+2εm)

In contrast, in the low angular frequency limit, the Clausius-Mossotti factor depends on electrical conductivities of both the particle and the liquid medium:

[9] lim(ω→0)fcm=(σp-σm)(σp+2σm)

### Dielectrophoretic force on the spherical shell

The dielectrophoretic force is used in different methods according to the literature. In a report by Li et al. (26), patterning micro-electrodes is within the microchannel by using photolithography, namely, a metal-electrode based DEP (eDEP). Consequently, metal electrodes are fabricated. In this method, it is necessary to apply the AC voltage to the electrodes because separation requires the electric field gradient. The main disadvantages of this method are the complexity of manufacturing and the chemical reactions of the electrodes. To tackle this problem, an alternative method is suggested, insulatorbased DEP (iDEP), where the direct voltage is used to create the electric field gradient. iDEP applies methods to create obstacles in the microchannel and uses curvature in the microchannel. Straight microchannels with electrode obstacles have some limitations such as the effect of excessive shear stress applied on the particles, joule heating effect, and creation of local nonuniform electric fields. If the insulating curved microchannels are used (i.e., circular, serpentine, spiral etc.), the limitations are relatively less. It should be noted that, in the alternative method, the applied voltage to the electrodes is direct and greater than the first method (19).

The eDEP-based separation methods have many applications in the fields of chemical and biochemical processes, such as separation and sorting of fine particles (i.e., proteins, carbon-nanotubes, viruses). iDEP-based sorting methods are proper for separation of bioparticles such as bacteria, RNA, and DNA (27). The structure of biological particles is more complex compared with solid homogeneous spherical particles. Therefore, the nonhomogeneous spherical particle model should be capable of calculating the DEP force exerted on the bioparticles. The concentric multi-shell model is widely used for calculation of the DEP force that acts on biological particles. In the simplest case, a cell is considered to be a spherical single-layer model. In this case, as shown in Figure S1 (See Supplementary Online Information at www. celljournal.org), the layered particle model is substituted as a homogenous particle with an equivalent radius and permittivity.

According to the description provided, the complex permittivity of particle, ${\tilde{\varepsilon }}_{p}$ is replaced with equivalent complex permittivity ${\tilde{\varepsilon }}_{p}$. Thus, the effective complex permittivity of ${\tilde{\varepsilon }}_{p}$ is substituted in the Clausius-Mossotti factor, which is defined as (24):

[10] fcm(ε~m,ε~pʹ)=(ε~pʹ-ε~m)(ε~pʹ+2ε~m)

Where (24):

[11] ε~pʹ=ε~1[(a3+2(ε~2-ε~1)(ε~2+2ε~1))(a3-(ε~2-ε~1)(ε~2+2ε~1))]

and 'a' is defined as R1/R2 .

Cellular interior conductivity and permittivity can be measured with the electrorotation method. In this method, the rotation of cells is measured under the influence of the torsional torque generated by the rotational electric field as a function of electric field frequency. In order to estimate the dielectric properties of cells, the measured spectral data is fitted to a curve using the optimization of single-shell model parameters (28). The Clausius-Mossotti factor can be calculated using the estimated dielectric properties of the cells. Table 1 lists the dielectric and physical properties of platelets, red blood cells, neutrophils, large lymphocytes and their suspension medium, including the cell diameter (d), membrane thickness (t), the specific membrane capacitance (C_mem_), the internal electrical conductivity (σ_int_), the internal relative permittivity (ε_rint_), the membrane electrical conductivity (σ_mem_), and the membrane relative permittivity (ε_mem_).

Figure S2 (See Supplementary Online Information at www.celljournal.org) shows the DEP spectra of spherical particles with a single-shell model for dielectric properties of blood cells using the medium with a conductivity of 55 mS/m, where the Clausius- Mossotti factor is computed in the MATLAB software with a single-shell model. The real part of the Clausius- Mossotti factor ranges from -0.5 to 1 and can increase or decrease the DEP forces. If the Clausius-Mossotti factor takes a positive sign (P-DEP), the particles are attracted to the higher electric field zone; if the factor takes a negative sign, the particles are attracted to the lower electric field zone (N-DEP).

When the applied electric field frequency is less than 100 (KHz), the Clausius-Mossotti factor for platelets, red blood cells, neutrophils, and large lymphocytes will approach -0.5 and, consequently, the blood cells experience N-DEP forces. According to equation [4], the dielectrophoretic force is proportional to the cube of the radius, and this results in the separation of blood cells in the downstream branches of the microchannel.

### Modelling and simulation

The medical importance of this study is to purify blood cells concentration at the microfluidic chip outlets to further biological investigations for diagnostic and therapeutic studies. The proposed separation method could be replaced by a fluorescence-activated cell sorter (FACS) for biomedical applications or differential cell counters in haematology analysis in medical laboratories. Furthermore, the designed chip can also be used as a home-use device for personalized medicine.

The schematic design of the microchip is shown in Figure S3 (See Supplementary Online Information at www.celljournal.org). There are two inlet branches and four exit branches in the design, which are connected to input and output reservoirs. The input reservoirs are for injection of buffer solution and samples into the microchannels "A" and "B", respectively. The length of the main microchannel is 1400 μm and the width of the microchannel is 300 μm. Microchannel "B" is 150 μm wide. The widths of the branch microchannels "C", "D", "E", and "F" are 120 μm, 100 μm, 120 μm and 100 μm, respectively. Each of the microchannels has a depth of 50 μm. The electrodes are placed on the right side of the microchannel. Electrodes 1 and 2 have a width of 170 μm and 40 μm, respectively. The electric fields can be calculated by the Laplace equation (equation [12]). In the mentioned schematic, the insulated microchannels are connected to the reservoirs and the electrodes that apply the AC electric fields on the passing fluid through the main microchannel.

[12] $$\nabla^{2}Ø=0$$

**Table 1:** The dielectric and physical properties of different blood cells and their suspension medium


Property	Platelets	Red blood cells	Neutrophils	Large lymphocytes	Suspension medium

Diameter (d, μm)	2.0 (18, 29)	6.2 (30)	10.0 (31, 32)	12.0 (32, 33)	-
Thickness (t, nm)	8 (34)	8 (35)	7 (36)	7 (36)	-
C_mem_ (mF/m^2^)	7.9 (34)	8 (36)	11 (37)	16.2 (38)	-
σ_int_ (S/m)	0.16 (34)	0.31 (39)	0.6 (37)	0.83 (38)	0.055
ε_rint_	50 (34)	59 (39)	150.9 (37)	73.2 (38)	78
σ_mem_ (S/m)	1e-7 (34)	1e-6 (39)	1.4e-7 (37)	1.4e-7 (38)	-
ε_mem_	7.2 (34)	4.44 (39)	8.7 (37)	12.8 (38)	-


Where: Ø is defined as the phasor of the alternating electrical potential applied on the electrodes.

In the proposed design (Fig .S3) (See Supplementary Online Information at www.celljournal.org), the Reynolds number is extremely low; therefore, the inertial term in the Navier-Stokes equation can be omitted, resulting in:

[13] $$\mu \nabla^{2}u=\nabla p$$

Where: u is the fluid velocity, ∇p is the pressure gradient, and μ is the dynamic viscosity. For the walls of the microchannels, we considered the no-slip boundary conditions. The flow velocities at the input "A" and “B” are fixed and the output flow at positions "C", "D", and "E" flows within the exited reservoirs with zero gauge pressure. We took into consideration the following assumptions for conducted simulation: i. The fluid flow has very low Reynolds number values (Re≪1) and the inertial term in the Stokes equations is dropped. Consequently, the fluid flow is considered a type of creeping flow, ii. The fluid flow is diluted to the extent that the effect of particle interaction is ignored and the particles do not exert any significant force on each other. In addition, the coupling between the fluid and particle phase is considered to be one-way. Thus, there is negligible particle force on the fluid, iii. The fluid flow is injected in the microchannel from branches "A" and "B" (Fig .S3) (See Supplementary Online Information at www.celljournal.org). The flow field is planar and thus the "z" component of the velocity is negligible compared with the two other components of the velocities, iv. The chip is installed horizontally. Consequently, the gravitational force can only cause particle deposition, v. The walls of the microchannels and the particles are not porous. Therefore, the effect of the particles and the walls are considered impermeable solids, and vi. The thermal gradient inside the microchannels is relatively low and does not affect the particles and fluid velocities.

The displacement changes of the particles can be calculated by time integrating the particles velocity, as:

[14] $$x_{p}(\tau )=x_{0}+\int u_{p}(t)\mathrm{dt}$$(0 ≪ t ≪τ)

Where: x_p_ (τ) is the location of the microchannel liquid outlet at the discharge hoses, x_0_ is the initial position of the particle, and u_p_ (t) is the particle velocity.

According to Newton’s law, the translational motion of a particle is explained by (24):

[15] Fext=mp(dupdt)

Where: F_ext_ is defined as the total of the superficial and volumetric forces applied to the particles and m_p_ is considered the particle mass.

The exerted drag force on a spherical particle is calculated at a very low Reynolds number (Re≪1) by the following relation according to Stokes low (40):

[16] $$F_{\mathrm{drag}}=3\mathrm{\pi \mu d}(u-u_{p})$$

Where: μ is the dynamic viscosity, d is the particle diameter, u is the fluid velocity, and u_p_ is the particle velocity within the fluid.

In the conducted design, it is assumed that the particles move across the microchannels at a constant velocity. Substituting equations [16] and [4] into equation [15], we calculate the particle velocity as:

[17] up=u-(εmRe[fcm]R2∇Erms2)3μ

## Results

### The particle separation that resulted from the dielectrophoresis force

Separation of platelets, red blood cells, neutrophils, and large lymphocytes was simulated by the DEP fieldflow- fractionation in COMSOL Multiphysics software (version 5.1; *https://www.comsol.de/products*). Both forces of the flow focusing and the DEP affected the particles’ trajectories and resulted in separation of the cells according to size. The prepared sample and buffer solution are injected into the downstream branches. Using the applied hydrodynamic pressures on the inlet solutions, the cells focused on the right side of the microchannel and, at the same time, the dielectrophoretic voltage is applied to the electrodes, which resulted in separation of the particles.

**Fig 1:**
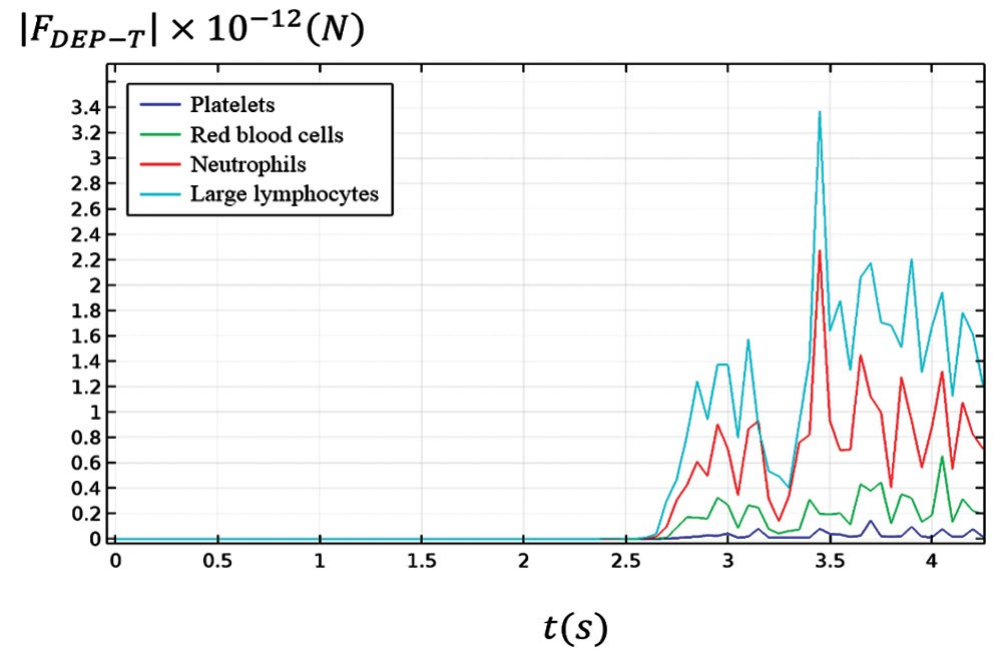
Comparison of the applied dielectrophoretic forces on platelets, red blood cells, neutrophils, and large lymphocytes.

Figure 1 shows the magnitude of the exerted dielectrophoretic forces on the blood cells and the resultant fluctuations of dielectrophoretic forces from the alternating electric field can be seen in this figure.

The slope of the beginning of the graph is almost zero, whereas the slope of the end gradually decreased because in these areas the effect of the electric field decreased compared with surrounding areas and in a time decreasing nature. The maximum slope of the diagram occurred during the time interval of 3.4 to 3.5 seconds because, in this interval, the particles experienced the maximum electric field gradient

**Fig 2:**
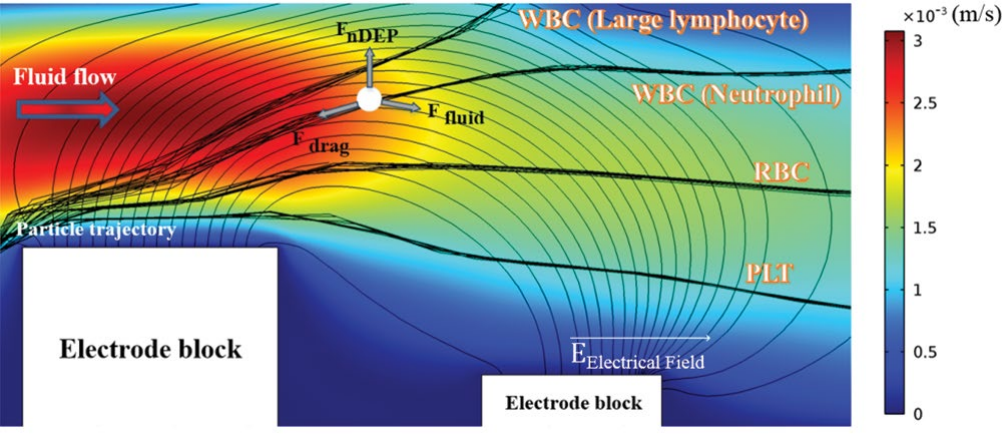
Electric field streamlines, force diagram, and fluid velocity distribution near the electrode blocks, where the blood cells are repelled of zone with higher electric field and are carried by the hydrodynamic force exerted by fluid.

We used the proposed design for continuous separation of the cells, where the localized AC-DEP forces were applied to cells located around the electrode blocks. Figure 2 shows the electric field streamlines, exerted forces diagram on the cell, and velocity magnitude distribution near the electrodes for a cell that passed through the microchannels. The particles were followed by the fluid flow streamlines because the electrodes did not apply the alternating electric field to the microchannels. In contrast, since an electric potential was imposed on the electrodes, the electric field gradient was created by the electrodes around the corners of the electrode blocks, which resulted in the generation of the DEP forces. In this design, the nDEP forces were exerted on the cells, which caused them to be repelled from the corners of the electrodes. Among the leaded cells, the white blood cells had the largest size and experienced the greatest nDEP force magnitude, and were conducted to outlet channels "E" and "F". The red blood cells and the platelets moved through the outlet channels "D" and "C" respectively, and experienced the nDEP forces proportional to their volumes.

Figure S4 (See Supplementary Online Information at www. celljournal.org) shows the convergence plot of the absolute error versus iteration number. The validation process is performed for the steady-state condition of the fluid flow. The Newton-Raphson method is used to correct the absolute error terms of the momentum equations. To solve the equations, the Newton-Raphson method starts with an initial guess and continues to converge the obtained answer to the exact answer with the desired precision. In Figure S4 (See Supplementary Online Information at www.celljournal.org), we show the absolute error plot of the fluid flow equation solutions with an absolute error of 0.85, which continued until it converged to an absolute error less than 10^-13^.

In Table S2 (See Supplementary Online Information at www.celljournal.org), the applied drag force on the neutrophils is calculated in the middle zone of the microchannel (t=2.85 seconds after injection of the particles). The results showed that magnitude of the applied drag force on the neutrophils was independent of mesh size for the normal to the fine grid sizes with an uncertainty of 1 e -11 N. The magnitude of the drag force was equal to 1.758 e -10 on average.

**Fig 3:**
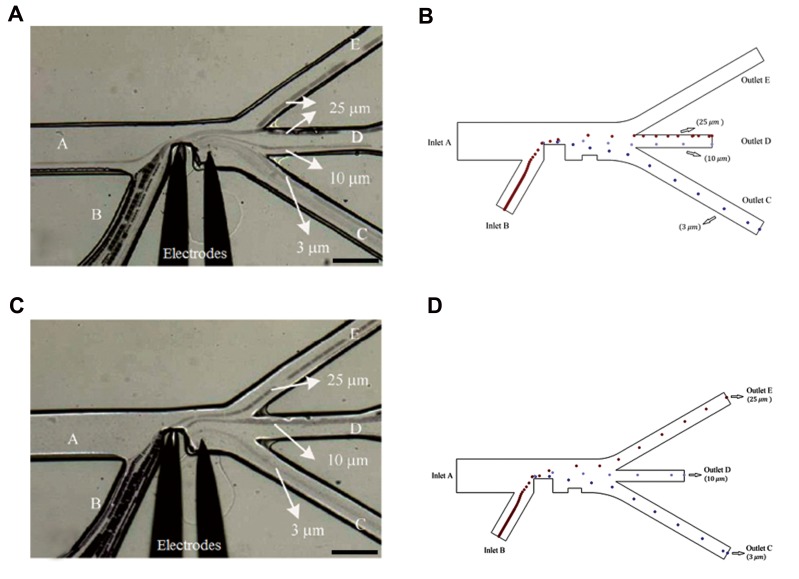
A comparison of the presentation model in this manuscript with the presentation model by Ye et al. (21). **A.** The path lines of three size particles for voltage of 112.5 V (experimental data reported by Ye et al. (21)), **B.** Prediction of the path lines of three size particles for voltage of 112.5 V (simulated data is reported in this article), **C.** The path lines of three size particles for voltage of 150 V (experimental data reported by Ye et al. (21)), and **D.** Prediction of the path lines of three size particles for voltage of 150 V (simulated data is reported in this manuscript).

We used the geometrical model applied by Ye et al. (21) to validate the simulation. The simulation showed that latex particles (polystyrene microspheres) with diameters of 3 μm, 10 μm, and 25 μm were conducted into different outlet channels. The path lines of the particles were compared with the experimental path lines presented in the reference article. Figure 3 shows a comparison of the two mentioned models, where the applied frequency to the electrodes equalled 1 MHz and flow velocity ratio between branch "A" and branch "B" (inlets) equalled 4.2. Of note, the applied alternating voltage on the electrodes could affect the magnitude of the applied dielectrophoretic force on the particles (Fig .3A-D).

The relative error with respect to the experimental data is expressed as:

[18] α=|(PA-PB)PA|×100

Where: P_A_ is the measured distance of the particles from the upper wall of the microchannel branches and P_B_ is the calculated distance.

Considering that in Figure 3A, the 25 μm particles did not cross through just one branch. Thus, in order to measure P_A_ in Figure 3A and P_B_ in Figure 3B, we took into consideration the upper walls of both outlets "E" and "D". According to our image processing, we assumed that in Figure 3A, 80% of particles with a diameter of 25 μm passed through outlet "D".

Table 2 shows the magnitude of α for different sizes of polystyrene microspheres with diameters of 25 μm, 10 μm, and 3 μm for the 112.5 V and 150 V voltages applied to the electrodes.

The results showed that the particle trajectory of the present work was similar to that reported by Ye et al. (21), with an acceptable deviation of 22.5%. This indicated that the presented model could be used in the separation applications. Therefore, we developed the model for 4 different size channels in order to separate the 4 blood cell types.

It is important that the particle separation be independent of the initial position of the released particles. We have taken this into consideration in the design and it could affect the separation precision. The released particles of the initial various locations were separated successfully (Fig.S5) (See Supplementary Online Information at www.celljournal.org). The density and viscosity values of the buffer solution are ρ_f_=1000 Kg/m^3^ and μ_f_=10^-3^ Pa.s, respectively. According to Figure S2 (See Supplementary Online Information at www. celljournal.org), the electric field frequency is considered to be 100 KHz. For this reason, the Clausius-Mossotti factor of the cells approached -0.5.

**Table 2:** The magnitude of α for the polystyrene microspheres with different diameters and the voltages of 112.5 V and 150 V (21)


Voltage applied to the electrodes	Polystyrene microsphere diameters (μm)	Measured distance of the particles from the upper wall (μm)	Calculated distance of the particles from the upper wall (μm)	Corresponding relative errors (%)	Average of the relative errors (%)	Overall average of the relative errors (%)

	25	13.0	9.5	26.9		
112.5 V	10	82.2	74.0	10.0	24.0	
	3	49.6	67.1	35.3		
						22.5
	25	57.8	41.6	28.0		
150.0 V	10	36.1	43.2	19.6	21.1	
	3	55.4	46.6	15.8		


**Fig 4:**
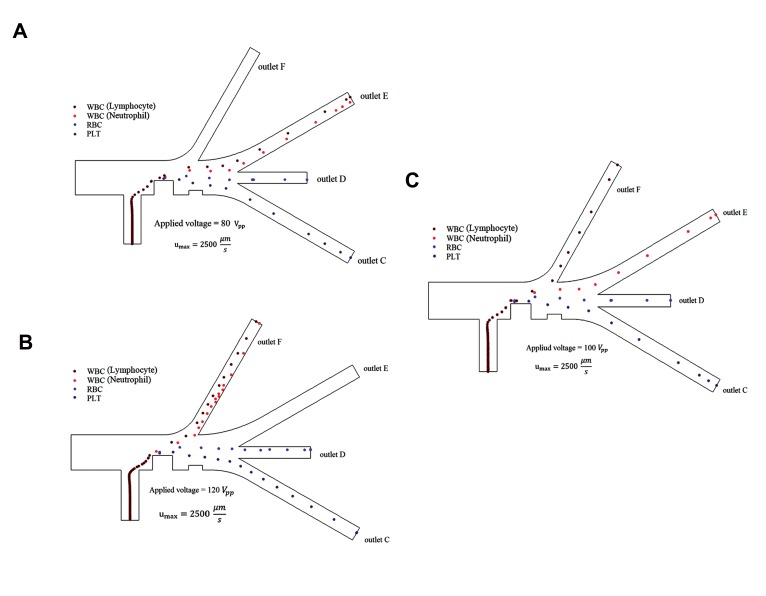
The blood cell trajectory when the applied voltage to the electrodes was: **A.** 80 V_pp_, **B.** 120 V_pp_, and **C.** 100 V_pp_.

We used the applied voltage to the electrodes to generate the gradient of the electric field intensity (∇E2) in the microchannel. The DEP forces were created by the gradient of the electric field intensity. Thus, in order to continuously separate the cells that had high purity, it was necessary to check the effect of the applied voltage on the cell separation process (Fig.4A-C). The electric field gradient in the corners of the electrodes was higher than at other places in the microchannels. Therefore, the particles experienced more DEP forces in the corners of electrodes, which resulted in cells that repelled from the corners. According to equation [4], the DEP force is a function of the gradient of the electric field intensity, the particle volume, and the real part of the Clausius-Mossotti factor. 

We used the applied voltage to the electrodes to generate the gradient of the electric field intensity (∇E2) in the microchannel. The DEP forces were created by the gradient of the electric field intensity. Thus, in order to continuously separate the cells that had high purity, it was necessary to check the effect of the applied voltage on the cell separation process (Fig .4A-C). The electric field gradient in the corners of the electrodes was higher than at other places in the microchannels. Therefore, the particles experienced more DEP forces in the corners of electrodes, which resulted in cells that repelled from the corners. According to equation [4], the DEP force is a function of the gradient of the electric field intensity, the particle volume, and the real part of the Clausius-Mossotti factor.

Thus, as the diameter and the applied voltage become higher, repelling will increase

Figure 4A-C shows separation of the blood cells. As depicted in Figure 4A, by applying voltage and pressure differences, the lymphocytes and the neutrophils were simultaneously removed from outlet "E", the red blood cells were removed from outlet "D", and the platelets were removed from outlet "C". This indicated that the particles did not experience successful separation from outlet "E" because the applied voltage was not enough to shift the larger particles further. Therefore, in this case, the performed separation was not successful.

In the next mode (Fig .4B), we applied a higher voltage to the electrodes. This applied voltage to the electrodes was 120 V_pp_ (peak-peak voltage). When the voltage was applied, the lymphocytes were shifted and removed from outlet "F" and the red blood cells were drawn toward the upper wall of the microchannel branch (branch "D"). This event increased the likelihood of the separation error.

Finally in the last mode, the applied voltage to the electrodes was 100 V_pp_. Figure 4C shows successful separation of the blood cells. The simulation results show that the proposed design has the ability to separate the blood cells based on cell size with high accuracy. For a successful separation, it is necessary for the DEP and drag forces to be properly employed.

## Discussion

We evaluated this design by simulating trajectories of large lymphocytes (diameter =12.0 μm) (32, 33), neutrophils (diameter =10.0 μm) (31, 32), red blood cells (d=6.2 μm) (30), and platelets (d=2.0 μm) (18, 29) in COMSOL Multiphysics software (version 5.1.) The COMSOL software is based on the finite element method for discretization of partial differential equations on the computational domain. In order to analyse the model, the finite element divide the model into small geometric zones. As the next step, polynomial functions were used to calculate the velocity and pressure components. At first, the alternating electric field and the fluid flow velocities were computed by considering the creeping flow and previously mentioned conditions, where the fluid flow has very low Reynolds number value (Re≪1). The suspending medium (the buffer solution) was diluted to the extent that, it was considered as water (ρ=997 kg/m^3^, μ = 0.9×10^-3^ kg/ms). The chip was installed horizontally. The flow field was planar and thus the "z" component of the velocity was negligible compared with the two other components of velocities. The walls of the microchannels and the particles were not porous and there was a negligible thermal gradient inside the microchannels. Finally, we simulated the particles’ trajectories in the microchannels. In the simulation, the samples were released from different positions in inlet "B".

## Conclusion

In this manuscript, we propose the four-branch model of the DEP method, taking into consideration the inherent properties of particles such as size, electrical conductivity, and permittivity coefficient. The presented design suggests a relatively simple setup to effectively separate 4 different blood cell types of 2.0 μm, 6.2 μm, 10.0 μm, and greater than 12.0 μm sizes. Therefore, it can be used to separate blood cells in different applications, including microfluidic separation devices and medical diagnostic processes. This device can separate blood cells using the single-stage system. The applied voltages to the electrodes can be adjusted such that shear stresses and joule heating effect are neglected. In the future, we suggest that the proposed device be extended for use in biomedical and diagnostic applications, with the goal of separating all blood components.

## Supplementary PDF


