# Supplementary material for: Blood Particle Separation Using Dielectrophoresis in A Novel Microchannel: A Numerical Study
Source: Cell J. 2019 Oct 14;22(2):218–26. doi: 10.22074/cellj.2020.6386 (PMC6874797; doi:10.22074/cellj.2020.6386)
Supplement: Supplementary file 1 [file Cell-J-22-218-s01.pdf]

www.celljournal.org

Supplementary Information for

Blood Particle Separation Using Dielectrophoresis in A Novel Microchannel: A Numerical Study

Omid Zahedi Siani, M.Sc.[1], Mahdi Sojoodi, Ph.D.[2]*, Mohammad Zabetian Targhi, Ph.D.[1]*, Mansoureh Movahedin, Ph.D.[3]

1. Faculty of Mechanical Engineering, Tarbiat Modares University, Tehran, Iran
2. Faculty of Electrical and Computer Engineering, Tarbiat Modares University, Tehran, Iran
3. Faculty of Medical Sciences, Tarbiat Modares University, Tehran, Iran

**Corresponding Addresses: P.O.Box: 14115-111, Faculty of Electrical and Computer Engineering, Tarbiat Modares University, Tehran, Iran*
P.O.Box: 14115-111, Faculty of Mechanical Engineering, Tarbiat Modares University, Tehran, Iran
Emails: sojoodi@modares.ac.ir, zabetian@modares.ac.ir

Table S1: Comparison of operation for various particles separation methods

Methods	Controllability	Efficiency	Operation	Damage	Cost	Reference
Microfluidic	Weak	High	Easy	Little	Low	(1)
Mechanical	Strong	Low	Hard	Large	Low	(1)
Optical	Strong	Low	Hard	Slight	High	(1)
Magnetic	Strong	Low	Hard	Slight	Low	(1)
Electrical	Strong	High	Easy	Slight	Low	(1)
Acoustic	Strong	High	Easy	Slight	Low	(2-4)

Table S2: Calculation of the applied drag force on the neutrophils versus the mesh size to validate the model and to find of the appropriate mesh size

Mesh size (number of the model elements)	The applied drag force magnitude on the NEUTROPHILs(N) at t=2.85 seconds
Extremely coarse (295)	0.557 e -10
Extra coarse (439)	2.237 e -10
Coarser (587)	1.550 e -10
Coarse (933)	2.010 e -10
Normal (1544)	1.480 e -10
Fine (2107)	1.865 e -10
Finer (3316)	1.930 e -10
Extra fine (8454)	0.950 e -10
Extremely fine (22592)	1.067 e -10

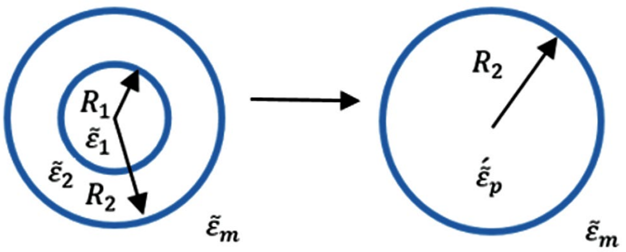

Fig.S1: The original two-layered particle is equivalent to a homogeneous sphere with an effective complex permittivity of $\tilde{\varepsilon}_{p.}$

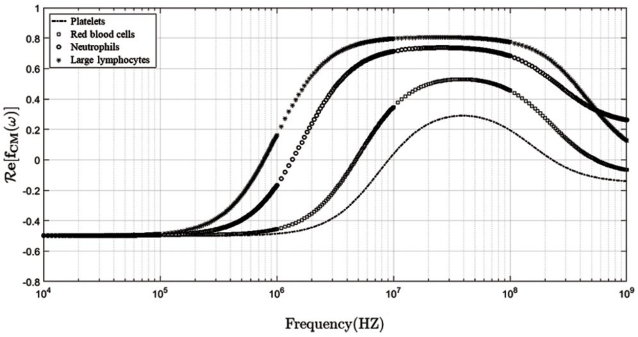

Fig.S2: The spectra DEP of the blood cells with single-shell model as a function of electric field frequency.

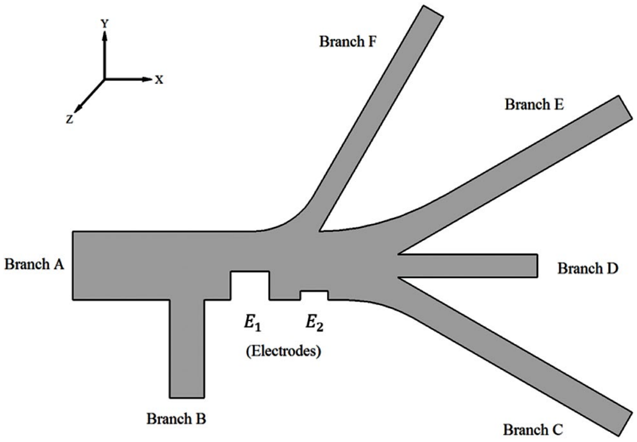

Fig.S3: Schematic of the PDMS microchip design with the employed electrodes in that.

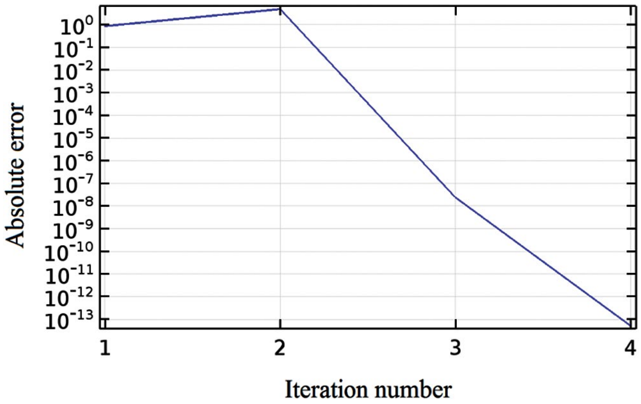

Fig.S4: The convergence plot of the absolute error versus iteration number for steady state condition of the fliud flow.

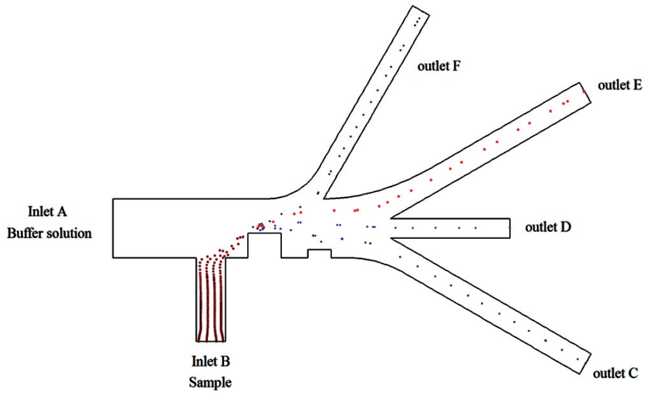

Fig.S5: The buffer solution was conducted from initial various locations of inlet "B". The buffer solution was also injected from inlet "A". The large lymphocytes, the neutrophils, the red blood cells, and platelets were guided from outlet "F", outlet "E", outlet "D", and outlet "C" respectively.

References

1. Qian C, Huang H, Chen L, Li X, Ge Z, Chen T, et al. Dielectrophoresis for bioparticle manipulation. Int J Mol Sci. 2014; 15(10): 18281-18309.
2. Guldiken R, Jo MC, Gallant ND, Demirci U, Zhe J. Sheathless size-based acoustic particle separation. Sensors (Basel). 2012; 12(1): 905-922.
3. Ma Z, Collins DJ, Ai Y. Detachable acoustofluidic system for particle separation via a traveling surface acoustic wave. Anal Chem. 2016; 88(10): 5316-5323.
4. Devendran C, Gunasekara NR, Collins DJ, Neild A. Batch process particle separation using surface acoustic waves (SAW): integration of travelling and standing SAW. RSC Advances. 2016; 6(7): 5856-5864.
